# T3 as predictor of mortality in any cause non-critically ill patients

**DOI:** 10.1530/EC-21-0080

**Published:** 2021-06-25

**Authors:** Erika Biegelmeyer, Iury Scanagata, Laura Alves, Murilo Reveilleau, Fernando Pereira Schwengber, Simone Magagnin Wajner

**Affiliations:** 1 Internal Medicine Division, Hospital de Clínicas de Porto Alegre, Internal Medicine Department, Universidade Federal do Rio Grande do Sul, Porto Alegre, Rio Grande do Sul, Brazil

**Keywords:** thyroid hormone, low T3 levels, non-critically ill patients

## Abstract

**Background:**

Low T3 syndrome refers to a set of thyroid hormone metabolism alterations present in the disease state. A correlation between low T3 and poor clinical outcomes in the intensive care unit is more established. Nonetheless, studies on non-critically ill patients are few and controversial.

**Objective:**

To evaluate the prevalence and predictive value of low T3 levels on 30-day and 6-month mortality in non-critically ill patients. Secondary outcomes evaluated the length of hospital stay, overall mortality, and hospital readmission.

**Design:**

Prospective cohort study.

**Methods:**

A total of 345 consecutive patients from the Internal Medicine ward of a tertiary hospital in southern Brazil were included and followed from October 2018 to April 2019 (6 months). Levels of total serum T3 were measured weekly, from admission to discharge, and correlated with 30-day and 6-month mortality.

**Results:**

Prevalence of low T3 was 36.6%. Low T3 levels were associated with higher 30-day hospital mortality (15.1% vs 4.1%, *P* < 0.001) and higher 6-month overall mortality (31.7% vs 13.2%, *P* < 0.001). Total serum T3 at admission was an independent predictor of 30-day hospital mortality.

**Conclusion:**

Low T3 levels are a prevalent condition among non-critically ill patients, and this condition is associated with poor clinical outcomes in this population. Total serum T3 levels, alone or in association with other predictive scores, were demonstrated to be an easy and valuable tool for risk stratification and should be further employed in this setting.

## Introduction

Low T3 syndrome reflects alterations in thyroid hormone levels without modification in the pituitary-thyroid axis. The full set of alterations includes a rapid decrease in serum triiodothyronine (T3) levels, accompanied by increased reverse T3 (rT3), and a decrease in plasma thyroxine (T4) as the disease progresses. These alterations occur in approximately 50% of patients with several chronic or acute diseases, but the mechanisms behind them are poorly understood (1, 2). Moreover, it remains controversial whether this is an adaptive response to conserve energy or a pathologic state. In the acute phase of illness, low T3 levels appear to be caused predominantly by dysfunction in peripheral thyroid hormone metabolism, mediated by iodothyronine deiodinases types 1, 2, and 3 (D1, D2, and D3, respectively) (1, 3, 4). Previous studies show that the depletion of the intracellular stock of antioxidants (such as reduced glutathione) secondary to disease creates an unfavorable environment for deiodinase function, thus inhibiting the T4 to T3 conversion process. These metabolic changes seem to be associated with physiological and immunological alterations secondary to the underlying disease (3, 5, 6, 7, 8).

This pattern of thyroid hormone alterations was described in several scenarios of acute or chronic illness (9, 10, 11, 12, 13, 14, 15, 16). In addition, there is a significant correlation between altered thyroid hormone levels and the severity and duration of disease (9, 10, 11, 12, 17, 18, 19, 20). Several studies found an association between low T3 and disease severity. For instance, patients with lower T3 levels had higher TIMI score and more severe myocardial injury (21). It was as well associated with a higher GOLD category and respiratory insufficiency (22, 23) and advanced metastatic disease (24) compared with other patients with the same comorbidities and without low T3 levels (25, 26, 27, 28). Maldonado *et al.* described lower mean T3 levels in short-term non-survivors among critically ill patients (34 vs 60 ng/dL, *P* < 0.001) (18). Liu *et al.* found that 30-day mortality among patients admitted due to community-acquired pneumonia was higher in the presence of low T3 syndrome (9.5% vs 2.6%) (29).

While the correlation between low T3 levels and patient morbimortality is less controversial in the ICU context (4, 30), studies outside the ICU including a heterogeneous population are rare and inconclusive (31). It still remains to be elucidated whether such metabolic alterations are present in non-critically ill patients and, if so, whether they are associated with worse clinical outcomes. Therefore, this study aimed to evaluate the prevalence of low T3 levels on a sample of non-critically ill patients in an internal medicine ward and identify the risk of mortality associated with it. We hypothesize that low levels of T3 are prevalent in this population, and, when present, can be associated with higher mortality rates independent of the disease.

## Methods

This was a prospective cohort study, which included 345 consecutive patients admitted to the Internal Medicine ward at Hospital de Clínicas de Porto Alegre, a tertiary hospital in southern Brazil, from October 2018 to April 2019. Institutional Ethics Board Committee (Ethic board committee from Hospital de Clínicas de Porto Alegre) specifically approved these study protocols (IRB 5327-190062) and written informed consent was obtained from all patients. The primary goal was to determine the correlation between low levels of plasma T3 hormone at hospital admission and 30-day hospital mortality. Secondary outcomes included 6-month overall mortality, hospital length of stay, and hospital readmission. The study did not interfere with regular medical care.

A total of 697 consecutive patients were evaluated, 26 refused to participate, and 326 met the exclusion criteria previously established: 4 (1.1%) were under 18 years of age, 49 (13.9%) had chronic kidney disease under definitive RRT, 101 (28.7%) had previous hospital discharge 30 days prior to readmission, 10 (2.8%) had HIV infection with CD4 < 250 cells/mm^3^, 7 (2.0%) used immunosuppressive therapy, 61 (17.3%) had chronic thyroid disease, 9 (2.6) used lithium or amiodarone, while 84 (23.9%) were transferred from ICU. A total of 345 patients were included and followed-up for 6 months. Clinical, laboratory and anthropometric information were prospectively collected from medical charts. Causes of hospital admission are described in Table 1. All patients were contacted via phone call after 6 months of hospital discharge to evaluate health status and hospital readmission in this period. Patients were divided into two subgroups based on serum T3 level at admission: low T3 (≤75 ng/dL) and normal T3 (>75 ng/dL). Given the ongoing debate on the appropriate T3 level threshold for defining NTIS, the lower limit of normality for T3 level in the assay used was defined as the cutoff point (32). Venous blood samples were obtained, and total serum T3 levels were measured at admission (baseline), and weekly afterward, for up to 4 weeks or discharge. Serum T3 levels were measured using an immuno electrochemiluminescent assay (ADVIA Centaur XP – Siemens). Assays were performed in duplicate on batched serum samples and stored at −20°C until study completion. The interassay coefficients of variation were 7.1%. The intra-assay coefficients of variation were as follows: T3, 6.2–7%. Normal ranges of T3 were 75–180 ng/dL.

**Table 1:** Demographic, baseline clinical and laboratory characteristics.

	Total (345)	Low T3 (126)	Normal T3 (219)	P*
Causes of hospital admission				
Infection, *n* (%)	137 (39.7)	49 (38.9)	88 (40.2)	0.452
Cardiovascular disease, *n* (%)	35 (10.1)	14 (11.1)	21 (9.6)	0.391
Respiratory disease, *n* (%)	16 (4.6)	4 (3.2)	12 (5.5)	0.242
Gastroenterological disease, *n* (%)	28 (8.1)	12 (9.5)	16 (7.3)	0.297
Neurologic disease, *n* (%)	25 (7.2)	9 (7.1)	16 (7.3)	0.570
Acute kidney injury, *n* (%)	20 (5.8)	11 (8.7)	9 (4.1)	0.065
Neoplasia, *n* (%)	80 (23.2)	34 (27)	46 (21)	0.129
Bleeding, *n* (%)	12 (3.5)	2 (1.6)	10 (4.6)	0.123
Vascular emergency, *n* (%)	9 (2.6)	3 (2.4)	6 (2.7)	0.571
Autoimmune disease activity, *n* (%)	8 (2.3)	2 (1.6)	6 (2.7)	0.390
Other cause for admission, *n* (%)	7 (1.2)	0 (0)	7 (1.8)	0.127
Clinical and demographic data				
Age, mean ± s.d., years	60.5 ± 15.9	65.0 ± 14.0	57.9 ± 16.3	0.000
Female sex, *n* (%)	175 (50.7)	69 (54.8)	106 (48.4)	0.265
BMI, mean ± s.d., kg/m^2^	26.4 ± 6.9	25.8 ± 6.4	26.7 ± 7.2	0.365
Hypertension, *n* (%)	170 (49.3)	69 (54.8)	101 (46.1)	0.146
Type 2 diabetes mellitus, *n* (%)	95 (27.5)	47 (37.3)	48 (21.9)	0.003
Non-metastatic neoplasia, *n* (%)	55 (15.9)	22 (17.5)	33 (15.1)	0.647
Metastatic neoplasia, *n* (%)	70 (20.3)	31 (24.6)	39 (17.8)	0.164
Congestive heart failure, *n* (%)	53 (15.4)	20 (15.9)	33 (15.1)	0.877
Ischemic heart disease, *n* (%)	48 (13.9)	23 (18.3)	25 (11.4)	0.105
Cardiac arrhythmia, *n* (%)	43 (12.5)	17 (13.5)	26 (11.9)	0.735
Heart valve disease, *n* (%)	10 (2.9)	2 (1.6)	8 (3.7)	0.337
COPD, *n* (%)	41 (11.9)	16 (12.7)	25 (11.4)	0.732
Asthma, *n* (%)	13 (3.8)	3 (2.4)	10 (4.5)	0.388
Chronic renal failure, *n* (%)	59 (17.1)	32 (25.4)	27 (12.3)	0.006
Cerebrovascular disease, *n* (%)	41 (11.9)	18 (14.3)	23 (10.5)	0.304
Dementia, *n* (%)	23 (6.7)	7 (5.6)	16 (7.3)	0.656
Hepatic disease, *n* (%)	25 (7.2)	8 (6.3)	17 (7.8)	0.673
HIV infection, *n* (%)	16 (4.6)	4 (3.2)	12 (5.5)	0.430
Rheumatologic disease, *n* (%)	18 (5.2)	9 (7.1)	9 (4.1)	0.314
Charlson, median (IQR)	5 (2–7)	5 (3–8)	3 (2–6)	0.000
APACHE II, median (IQR)	9 (6–12)	9 (7–13)	8 (5–11)	0.000
Laboratory results				
Hb, mean ± s.d., g/dL	11.4 ± 2.7	10.5 ± 2.6	11.9 ± 2.7	0.000
Leukocytes, median (IQR), 1000 cells/mm^3^	8.8 (5.7–12.5)	9.1 (5.9–13.5)	8.2 (5.5–11.5)	0.117
Lymphocytes, median (IQR), cell/mm^3^	1356.1 (833.5–2022.1)	1096.2 (692.4–1614.7)	1545.4 (911.7–2109.2)	0.000
Platelets, median (IQR), 1000 cells/mm^3^	249 (177–319)	266 (191–367)	228 (167–295)	0.117
C reactive protein, median (IQR), mg/L	40.1 (9.7–104.8)	56.4 (17.8–147.1)	29.1 (5.4–84.5)	0.000
Creatinine, median (IQR), mg/dL	0.98 (0.73–1.73)	1.33 (0.88–2.23)	0.88 (0.68– 1.16)	0.000
Urea, median (IQR), mg/dL	44 (30.7–69)	57 (39–101)	33 (25–51)	0.000
Albumin, mean ± s.d., g/dL	3.5 ± 0.8	3.18 ± 0.8	3.64 ± 0.7	0.000
Bicarbonate, mean ± s.d., mEq/L	23.3 ± 4.5	22.71 ± 4.64	23.74 ± 4.34	0.066
AST, median (IQR), U/L	24 (17–39)	19 (16–37)	28 (18–39)	0.032
ALT, median (IQR), U/L	21 (13–37.5)	20 (13–37)	21 (15–38)	0.272
AKL, median (IQR), U/L	121 (82–175)	133 (93.7–196.5)	112 (76.5–165)	0.088
GGT, median (IQR), U/L	73 (37–184)	118 (45–238)	66 (34–144.75)	0.308
Total bilirubin, median (IQR), mg/dL	0.4 (0.3–0.7)	0.4 (0.3–0.8)	0.4 (0.3–0.7)	0.423
Lactate, median (IQR), mmol/L	1.57 (0.9–2.3)	1.7 (1.0–2.48)	1.26 (0.91–2.18)	0.385

*P value for comparison between means or medians, using Student's *t*-test or Mann–Whitney *U-*test, according to normality of distribution.

AKL, alkaline phosphatase; ALT, alanine aminotransferase; AST, aspartate aminotransferase; Charlson, Charlson comorbidity index; GGT, gamma-glutamyl transpeptidase.

Comparisons between variables were performed using Student's *t*, Mann–Whitney *U*, Chi-square or Fisher’s exact tests. Serum T3 levels through hospital stay were compared by generalized estimated equations. Kaplan–Meier curves with log rank tests were performed for 30-day hospital survival and 6-month overall survival. Univariate followed by multivariate Cox regression was performed to identify independent predictors of 30-day hospital mortality. ROC curves were built to the evaluate accuracy of 30-day hospital mortality and 6-month overall mortality predictors. All statistical analysis was analyzed using Statistical Package for Social Sciences (SPSS) version 23 software (SPSS Inc.), with a significance of *P* < 0.05.

## Results

Baseline study sample characteristics are listed in Table 1. Average age was 60.5 years. There was no difference between gender distributions (50.7% female). Most common causes of hospital admission were infection of any site (37%). Most frequent comorbidities were hypertension (49.3%), type 2 diabetes (DM2) (27.5%), metastatic neoplasia of any system (20.3%), and chronic renal failure (CRF) (17.1%). A total of 126 patients (36.6%) had low T3 at admission (time 0). Low T3 patients were older (mean age 65.0 ± 14.0 vs 57.9 ± 16.3, *P* < 0.001), had higher rates of type 2 diabetes mellitus (37.3% vs 21.9%, *P* = 0.003), and chronic renal failure (25.4% vs 12.3%, *P* = 0.006). No difference was observed on the causes of hospital admission between low and normal T3 groups. Clinical scores were higher in the low T3 group (Charlson: median 5, IQR 3–8 vs median 3, IQR 2–6, *P* < 0.001; APACHE II: median 9, IQR 7–13 vs median 8, IQR 5–11, *P* < 0.001). Low T3 group had lower hemoglobin levels (10.5 ± 2.6 g/dL vs 11.9 ± 2.7 g/dL, *P* < 0.001), total lymphocyte count (median 1096.2 cell/mm^3^, IQR 692.4–1614.7 cell/mm^3^ vs median 1545.4 cell/mm^3^, IQR 911.7–2109.2 cell/mm^3^, *P* < 0.001), albumin (3.18 ± 0.8 g/dL vs 3.64 ± 0.7 g/dL, *P* < 0.001), and aspartate aminotransferase (AST) (median 19 U/L, IQR 16–37 U/L vs median 28 U/L, IQR 18–39 U/L, *P* = 0.032). They also had statistically higher levels of C-reactive protein (CRP) (median 56.4 mg/L, IQR 17.8–147.1 mg/L vs median 29.1 mg/L, IQR 5.4–84.5 mg/L, *P* < 0.001), creatinine (Cr) (median 1.33 mg/dL, IQR 0.88–2.23 mg/dL vs median 0.88 mg/dL, IQR 0.68–1.16 mg/dL, *P* < 0.001), and urea (Ur) (median 57 mg/dL, IQR 39–101 mg/dL vs median 33 mg/dL, IQR 25–51 mg/dL, *P* < 0.001).

**Figure 1:**
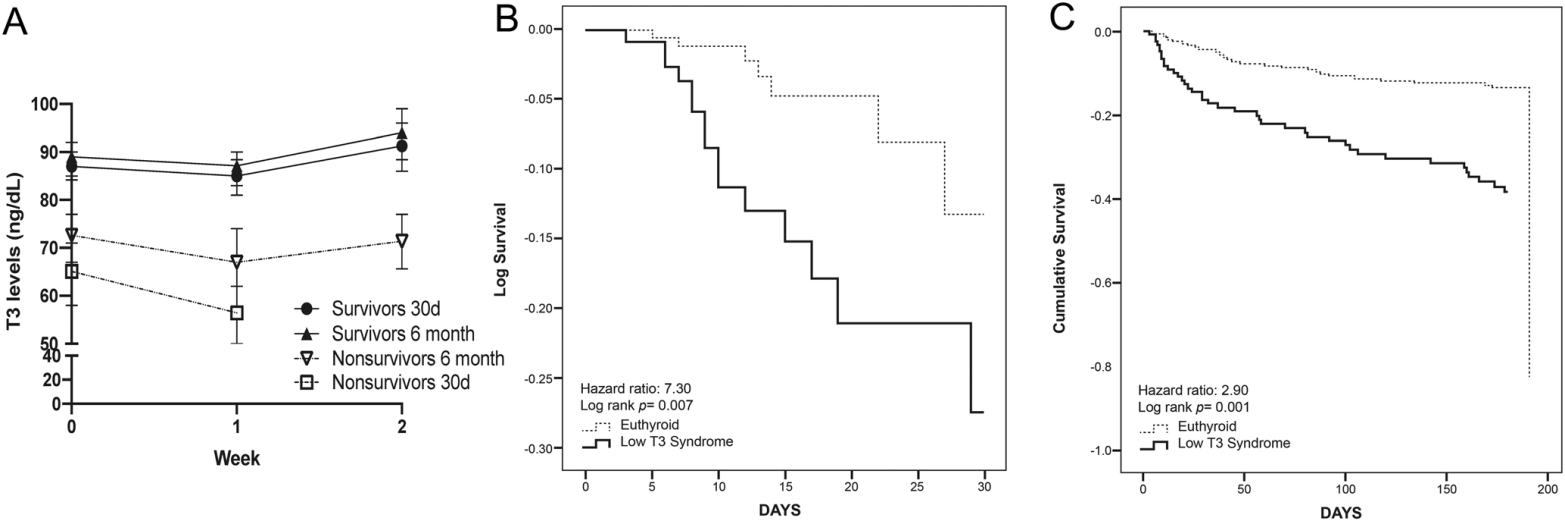
T3 levels and its effect on patient survival. (A) Mean T3 levels in 30-day and 6-month survivors and non-survivors. (B) ROC curve on 30-day survival and (C) ROC curve on 6-month survival of euthyroid and patients with low T3 levels.

Mean values of T3 throughout hospital stay are shown in Fig. 1A. Despite non-significant intra-group differences, there is a common pattern of decrease in T3 levels after the first week of admission. The 30-day mortality for the entire study sample was 8.1%, higher in the low T3 patients (15.1% vs 4.1%, *P* < 0.001). This difference is illustrated by Kaplan–Meier curves in Fig. 1B (Hazard ratio 7.3, log rank *P* = 0.007). Total 6-month overall mortality was 25.8%. Low T3 group also presented a higher 6-month overall mortality (31.7% vs 13.2%, *P* < 0.001) compared to the normal T3 group, as illustrated by Fig. 1C (Hazard ratio 2.9, log rank *P* < 0.001). Length of hospital stay was not different between the groups (median 12 days, IQR 8–19.6 days vs median 9 days IQR 6–14 days, *P* = 0.515). Readmission rate calculated as the percentage of readmitted patients within 6 months of discharge. This result was similar between groups (49.2% vs 42.4%, *P* = 0.261). However, low T3 patients had a shorter readmission-free period (median 30 days, IQR 10–90 vs 51 days, IQR 25–117, *P* = 0.042).

Univariate followed by multivariate Cox logistic regression analysis was performed to identify independent predictors of 30-day hospital mortality and identify possible confusion factors. Data on univariate analysis are detailed in Table 2. Two subsequent multivariate logistic Cox regression analyses were performed, as presented in Table 3. Model 1 included laboratory tests results and identified albumin and T3 levels at admission to be significant independent predictors of 30-day mortality. Model 2 included demographic and baseline clinical characteristics and identified age, metastatic neoplasia, chronic liver disease, and low T3 levels at admission to be significant independent predictors of 30-day hospital mortality.

**Table 2:** Univariate hazard ratios of variables for predicting 30-day hospital mortality.

	*P*	HR	95,0% CI for HR	
			Lower	Upper
Demographic and clinical characteristics				
Age	0.006	1.047	1.013	1.082
Sex	0.848	0.921	0.399	2.129
T3 at admission	0.011	3.225	1.312	7.927
Chronic renal failure (CRF)	0.172	4.054	0.545	30.172
Congestive heart failure	0.256	24.922	0.097	–
COPD	0.311	0.571	0.193	1.689
Ischemic heart disease	0.621	1.443	0.337	6.180
Arrhythmia	0.156	0.455	0.153	1.350
Non-metastatic neoplasia	0.725	1.244	0.368	4.206
Metastatic neoplasia	0.001	4.484	1.913	10.510
Chronic liver disease	0.344	0.555	0.164	1.880
HIV non-AIDS	0.520	21.577	0.002	–
Type 2 diabetes mellitus	0.302	0.632	0.265	1.509
Uncompensated DM2	0.997	1.002	0.296	3.392
Cerebral vascular disease	1.000	1.000	0.233	4.290
Dementia	0.763	1.363	0.182	10.194
Hypertension	0.055	2.504	0.979	6.406
Causes of hospital admission				
Admission due to infection	0.170	0.498	0.183	1.350
Admission due to cardiovascular disease	0.675	0.650	0.087	4.865
Admission due to respiratory disease	0.521	0.046	0.000	558.851
Admission due to gastroenterologic disease	0.757	1.259	0.294	5.394
Admission due to neurologic disease	0.470	0.046	0.000	198.453
Admission due to acute kidney injury	0.702	1.329	0.310	5.692
Admission associated with neoplasia	0.001	4.468	1.804	11.064
Laboratory tests				
Hemoglobin	0.008	0.808	0.691	0.945
Leukocytes	0.765	0.990	0.924	1.060
Lymphocytes	0.022	0.993	0.986	0.999
Platelets	0.116	0.997	0.994	1.001
C reactive protein	0.268	1.002	0.998	1.006
ESR	0.287	1.017	0.986	1.050
Creatinine	0.512	0.898	0.652	1.238
Urea	0.553	1.002	0.996	1.008
Albumin	0.000	0.258	0.136	0.488
Bicarbonate	0.051	0.913	0.833	1.000
AST	0.001	1.003	1.001	1.004
ALT	0.202	1.001	0.999	1.003
AKL	0.003	1.002	1.001	1.003
GGT	0.067	1.001	1.000	1.002
Total bilirubin	0.000	1.161	1.085	1.243
Lactate	0.745	1.047	0.794	1.380
Clinical scores				
APACHE II	0.000	1.097	1.047	1.149
CHARLSON	0.002	1.194	1.070	1.334

**Table 3:** Multivariate logistic regression analysis for independent predictors of 30-day hospital mortality.

	*P*	Exp(B)	95% CI for exp(B)	
			Lower	Upper
Model 1 laboratory tests				
Age	0.088	1.033	0.995	1.072
T3 at admission	0.034	0.969	0.941	0.998
Hemoglobin	0.177	0.831	0.636	1.087
Lymphocytes	0.092	0.994	0.988	1.001
Creatinine	0.106	0.653	0.389	1.095
Albumin	0.017	0.356	0.153	0.829
C reactive protein	0.277	0.996	0.990	1.003
Model 2 clinical conditions				
Age	0.023	1.060	1.008	1.114
Low T3 at admission	0.032	3.106	1.103	8.748
Congestive heart failure	0.973	0.000	0.000	–
COPD	0.164	2.552	0.681	9.563
Ischemic heart disease	0.710	0.737	0.148	3.678
Arrhythmia	0.138	2.899	0.711	11.818
Heart valve disease	0.676	1.603	0.175	14.660
Metastatic neoplasia	0.001	5.294	2.004	13.987
Chronic liver disease	0.038	4.879	1.095	21.675
Type 2 diabetes mellitus	0.286	1.710	0.638	4.581
Cerebral vascular disease	0.279	0.399	0.076	2.107
Chronic renal failure	0.123	0.190	0.023	1.573

**Figure 2:**
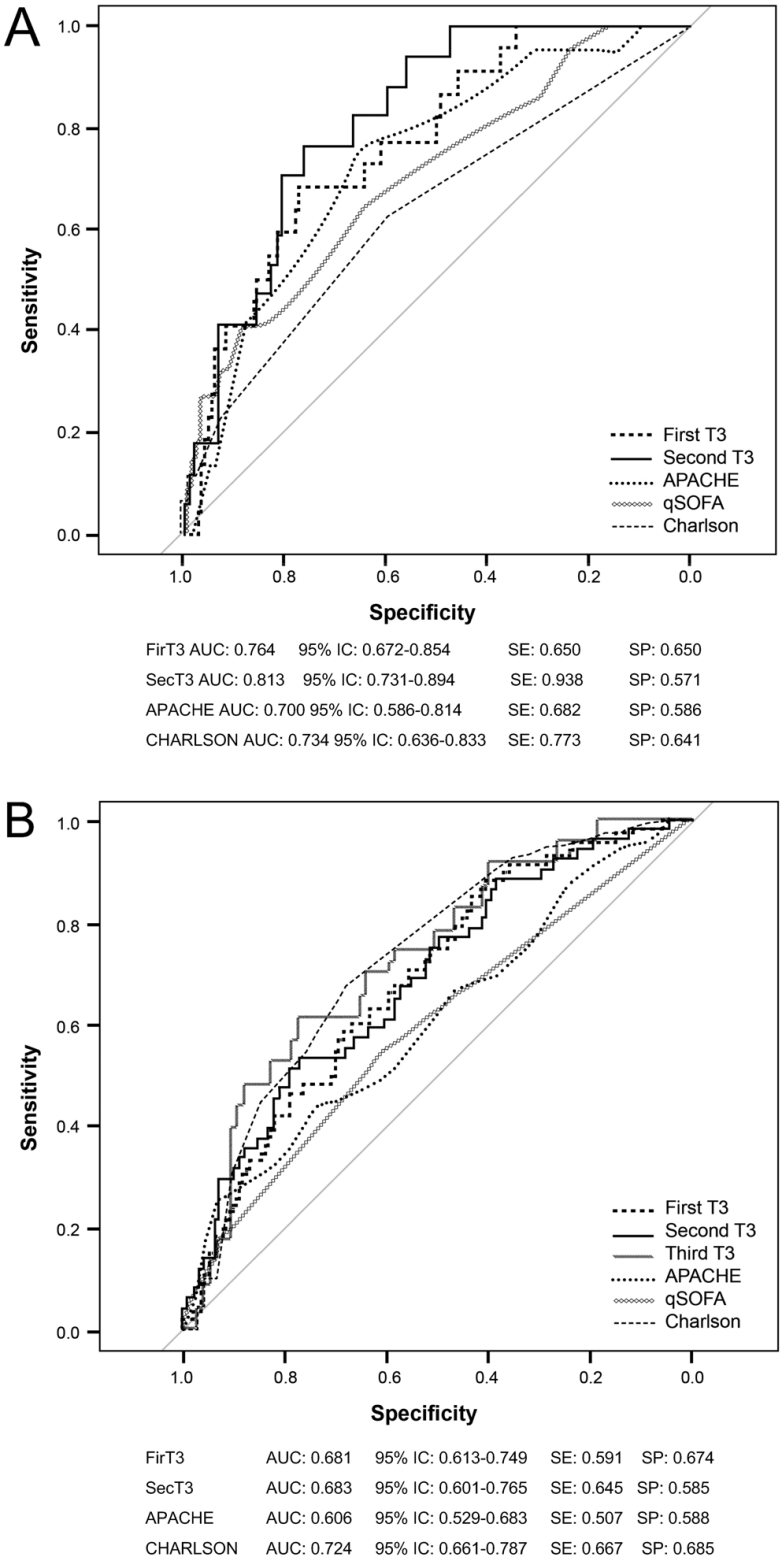
ROC curves comparing T3 and standard prognostic scores. (A) ROC curve comparing T3 and prognostic scores on 30-day hospital mortality. (B) ROC curve comparing T3 and prognostic scores on 6-month overall mortality. AUC, area under the curve.

A receiver operating characteristic curve analysis (ROC curve) was performed to assess T3 level accuracy in predicting 30-day and 6-month overall mortality, as compared to already established clinical prediction scores. Data are shown in Fig. 2A and B, respectively. Variables evaluated were T3 at admission (time 0), T3 after 1 week of hospital stay (time 1), APACHE II score, and Charlson score. Area under the curve (AUC) values for predicting 30-day mortality were higher for T3_time 1 (0.813, 95% IC: 0.731–0.894, *P* < 0.001), followed by the T3_time 0 (0.764, 95% IC: 0.672–0.854, *P* < 0.001). Subsequent bootstrap test identified no difference between T3_time 0, T3_time 1, APACHE II, and Charlson. T3_time 1 had the higher sensitivity to predict 30-day mortality but with less specificity (0.938 and 0.571, respectively). Area under the curve (AUC) values for 6-month overall mortality were higher for Charlson (0.724, 95% IC: 0.661–0.787), followed by T3_time 1 (0.683, 95% IC: 0.601–0.765), and T3_time 0 (0.681, 95% IC: 0.613–0.749), all with *P* < 0.01. Subsequent bootstrap test identified no difference among variables evaluated.

## Discussion

The present study identified low T3 levels in about one-third of patients admitted to the internal medicine ward. Low T3 levels were also associated with lower 30-day and 6-month survival rates, and with a shorter readmission-free period. T3 level was shown to be an accurate predictor of mortality, acting as an independent risk factor for 30-day mortality. Moreover, T3 levels were persistently lower in non-survivors throughout the hospital stay, whereas both T3 levels at admission and after 1 week were accurate in predicting mortality. This can represent an important and straightforward tool to help to identify patients at risk of higher morbidity, contributing to the decision-making process once a patient is admitted, regardless of the disease.

Low T3 syndrome mainly develops due to changes in peripheral thyroid hormone metabolism (1, 2, 3, 4, 33). While this phenomenon’s underlying mechanisms remain to be fully understood, the elevation of cytokine levels (TNF, INFa, IL1, and IL6) is considered the most significant mediators due to increased pro-inflammatory states secondary to the insult and present in different intensities in diverse pathological scenarios (6, 7). Low thyroid hormones interact negatively with several hemodynamic parameters and are believed to aggravate cardiac remodeling and heart failure progression, among several other alterations (34, 35, 36). Low T3 levels were once seen and interpreted as an adaptive response of metabolism to acute and chronic critical illness to reduce and preserve energy for vital functions. Nonetheless, ours as well as other studies suggest that the severity of low T3 levels is correlated with the progression of the disease, pointing to a maladaptive response, which can result in a hypometabolic state, among other alterations, helping perpetuate the underlying pathological process (3, 37).

Most studies in this field took place in intensive care units (18, 19, 20, 38). This work is original since it is the first cohort performed with heterogeneous patients in a general internal medicine ward to evaluate the prognostic role of T3. The prevalence of low T3 levels of 36.6% in our population is on the range of other small cohorts of specific diseases, reported to be of 10.4–71.9% (16, 24, 29, 39, 40, 41, 42). Interestingly, our approach of any cause admission patients also brought similar results with previous reports on 30-day mortality in patients with low T3 levels. During long-term follow-up of chronically ill patients, Ataoğlu *et al.* found low free T3 to be associated with a 56.1–122.4% increase in mortality (31). Interestingly, our study found that 6-month increased mortality associated with low T3 levels similar to patients on intensive care (31.7% vs 13.2%). The prospective design of our study reinforces the deleterious association of low levels of T3 with short- and long-term mortality.

Since our patients were heterogeneous regarding the primary disease, we assessed the severity of the disease through APACHE II and Charlson comorbidity index scores. Although both scores show to be significantly higher in the low T3 group, uni- and multivariate analysis show that those scores were not confounding variables, confirming an association between the severity of the primary disease and the presence of low T3. Since low T3 levels were observed despite the cause of admission, one putative reason could be mostly because of the inflammatory pattern of diseased patients. Notably, the T3-time 1 was a good prognostic marker as baseline T3. Of utmost importance, our results show that it is not necessary to know previous T3 levels nor take serial measures in order to establish the risk ratio of one specific patient and its statistic value persists for over a week after admission. T3 levels both on time 0 (admission) or time 1 had a good accuracy to predict short- and long-term mortality, comparable to other prognostic validated clinical scores. Accordingly, it was previously suggested that adding T3 to other severity scores (9, 29, 41). Moreover, serum T3 is an easy, reliable, and applicable test that can, individually or in combination with the other severity scores, increase the sensitivity to highlight those patients with a worse prognosis.

The findings shown here are of direct importance in clinical practice to help physicians identifying those patients that need more attention and a quick decision-making process, besides promptly helping on subjective medical decisions. We also observed a higher mortality on low T3 patients in a period of 30 days (HR 7.3), which is in agreement with previous studies (43, 44, 45). Even though the association of low T3 with long-term mortality is less clear, it has been observed increased mortality with HR of 1.62–2.13 in patients with lower T3 (44, 45), with this effect being attenuated by each year of follow-up (44). These data are similar to our 6-month follow-up (HR 2.9). These findings corroborate the importance of T3 as a predictor of poor prognosis in non-critically ill patients. We also observed a shorter readmission-free period on low T3 patients, but no difference was detected in the length of stay or readmissions rate in the 6 month period. Other studies found a longer length of stay in patients in ICU (15, 17). Besides, considering that the comparison is made with ICU patients, our finding suggests that low T3 levels could be seen as a marker of the severity of the disease as well as the metabolic alterations caused by it.

In order to determine whether low T3 levels could serve as an independent risk factor of mortality, we performed two distinct models of multivariate analysis since laboratory tests were already counted on clinical scores. Interestingly, both models demonstrated that T3 levels are an independent and an accurate marker of 30-day hospital survival. This was the most trustworthy way to analyze the number of variables we had. Other independent factors were low albumin, age, metastatic neoplasia, chronic liver disease (Child-Pugh C cirrhosis excluded). As observed by others, we also had an important prevalence of metastatic neoplasia patients, frequently malnourished, which could also be related to low albumin levels (25, 26, 27, 28), uni and multivariate analysis show that those features were not confounding variables, confirming an association between the severity of the primary disease and the presence of low T3 levels.

Our study has some limitations. First, this is a single-center study. Secondly, since the scope of this study was to determine the role of T3 in mortality prediction, we did not classify patients according to disease severity, such as ejection fraction percentage of heart failure and chronic renal failure stages. Thirdly, missing T3 samples resulted in little data on third and fouth weeks. Fourthly, some laboratory data were often missing, which resulted in multivariate analysis restrictions and a need for two analytic models.

Treatment of low T3 has proven to be non-efficient so far in preventing poor outcomes (46, 47, 48). However, some investigators suggest that it might be possible to reverse the primary cause of thyroid hormone metabolism dysfunction in specific populations, either by hormonal supplementation or by correction of the underlying disease metabolic alterations (49, 50, 51, 52, 53, 54, 55). The results here reported acknowledged that T3 levels impact the prognosis of general hospitalized patients. This extends to non-critically ill patients the benefits that low T3 levels represent both a prognosis marker and a potential therapeutic target. Further studies should identify subpopulations in which low T3 levels are more closely associated with outcome prediction and that would benefit with treatment.

## Conclusion

In conclusion, this study demonstrates that low T3 is prevalent in a heterogeneous cohort of general ward patients. Importantly, it acts as a reliable indicator of poor prognosis and mortality. Low levels of T3 on admission are associated with higher scores of severity and shorter readmission-free period regardless of the disease or previous T3 levels.

## Declaration of interest

The authors declare that there is no conflict of interest that could be perceived as prejudicing the impartiality of the research reported.

## Funding

This work was supported by FIPE/HCPA – Hospital de Clínicas de Porto Alegre Research Incentive Fund (190062) and CNPq – National Council for Scientific and Technological development (301585/2018-0).
